# A cross-sectional study on metabolic similarities and differences between inpatients with schizophrenia and those with mood disorders

**DOI:** 10.1186/s12991-020-00303-5

**Published:** 2020-09-22

**Authors:** Yoriyasu Uju, Tetsuto Kanzaki, Yuki Yamasaki, Tadayuki Kondo, Hideki Nanasawa, Yu Takeuchi, Yuta Yanagisawa, Shun Kusanishi, Chieko Nakano, Tetsuro Enomoto, Akahito Sako, Hidekazu Yanai, Shunichi Mishima, Seisuke Mimori, Kazuei Igarashi, Tsuyoshi Takizawa, Tatsuro Hayakawa

**Affiliations:** 1 Department of Psychiatry, Kohnodai Hospital, National Center for Global Health and Medicine, 1-7-1, Kohnodai, Ichikawa, Japan; 2 Department of Drug Informatics, Graduate School and Faculty of Pharmaceutical Sciences, Chiba University, 1-8-1, Inohana, Chuo-ku, Chiba, Japan; 3 Department of Internal Medicine, Kohnodai Hospital, National Center for Global Health and Medicine, 1-7-1, Kohnodai, Ichikawa, Japan; 4 Department of Diabetes, Endocrinology and Metabolism, Kohnodai Hospital, National Center for Global Health and Medicine, 1-7-1, Kohnodai, Ichikawa, Japan; 5 Department of Clinical Medicine, Faculty of Pharmacy, Chiba Institute of Science, 15-8, Shiomi-cho, Choshi, Japan; 6 Amine Pharma Research Institute, Innovation Plaza at Chiba University, 1-8-1, Inohana, Chuo-ku, Chiba, Japan; 7 Department of Biostatistics, Faculty of Pharmacy, Chiba Institute of Science, 15-8, Shiomi-cho, Choshi, Japan

**Keywords:** Schizophrenia, Mood disorders, Estimated glomerular filtration rate (eGFR), Smoking, Silent brain infarction (SBI), Dyslipidemia, Diabetes

## Abstract

**Background:**

One of the main causes of death in psychiatric patients is cardiovascular diseases which are closely related with lifestyle-related diseases. Psychiatric disorders include schizophrenia and mood disorders, whose symptoms and treatment medicines are different, suggesting that they might have different metabolic disorders. Thus, we studied the differences of lifestyle-related diseases between schizophrenia and mood disorders in Japan.

**Methods:**

This cross-sectional study was performed from 2015 to 2017. Study participants were 189 Japanese hospitalized patients (144 schizophrenia group, 45 mood disorders group) in the department of psychiatry at Kohnodai hospital. We examined physical disorders, metabolic status of glucose and lipid, estimated glomerular filtration rate (eGFR) and brain magnetic resonance imaging. We compared these data between schizophrenia and mood disorders groups using analysis of covariance or logistic regression analysis. In comparisons between inpatients with schizophrenia or mood disorders group and the standard, we quoted ‘The National Health and Nutrition Survey in Japan 2015’ by Ministry of Health, Labor and Welfare as the standard.

**Results:**

eGFR and prevalence of smoking were significantly lower in patients with mood disorder group than those with schizophrenia group by adjustment for age. In comparisons between patients with schizophrenia group or mood disorders group and each standard, the ratio of silent brain infarction (SBI) and cerebral infarction were significantly high in both groups. Schizophrenia group showed significantly higher prevalence of diabetes, low high-density lipoprotein (HDL) cholesterolemia, metabolic syndrome and smoking than the standard. Mood disorders group had significantly high prevalence of low HDL-cholesterolemia compared with the standard. Fasting blood glucose and HbA1c were significantly higher in schizophrenia group and female mood disorders group than the standard. Female mood disorders group had significantly decreased eGFR with increased ratio of eGFR < 60 ml/min than the standard.

**Conclusions:**

Participants of both groups had increased ratio of SBI and cerebral infarction, accompanied with glucose and lipid disorders. Compared with schizophrenia group, mood disorders group showed significantly low eGFR and prevalence of smoking.

## Background

It is well known that psychiatric patients have a short life expectancy. Henekens et al. [1] reported that schizophrenic patients have approximately 15 years shorter life time than general population and more than 60% of deaths are due to coronary heart diseases in the United States. In the countries from Europe, Asia, Australia, Africa and Japan predicted life time is 11–22 years shorter than the general population [2, 3]. Crump et al. [4] and Smith et al. [5] also reported that cardiovascular diseases and malignancy are the main causes of death in psychiatric patients, and cardiovascular diseases are likely to be underrecognized and undertreated in schizophrenic patients. These facts indicate that cardiovascular diseases are one of the most important causes of short life expectancy in psychiatric patients.

Diabetes, smoking, hypertension, dyslipidemia, visceral type obesity and chronic kidney disease (CKD) are risk factors of cardiovascular diseases. Indeed, there are many reports that schizophrenic patients have a high rate of diabetes, smoking, low HDL-cholesterolemia, obesity and metabolic syndrome [6–8]. Psychiatric patients tend to have unhealthy eating habits, shortage of exercise and smoking [8]. It is probable that these unhealthy lifestyles are related with increased risk factors of cardiovascular diseases. Furthermore, schizophrenic patients usually are administered typical or atypical antipsychotics. Halfdanarson et al. [9] reported that use of typical antipsychotics was decreased but that of atypical antipsychotics was elevated in the past 10 years globally. Some atypical antipsychotics cause adverse effects on glucose and lipid metabolism and induce diabetes and dyslipidemia [10, 11]. These side effects of antipsychotics also increase the risks of cardiovascular diseases.

Saku et al. [12] and Kondo et al. [3] reported that the standardized mortality rate of schizophrenic patients with heart disease was higher than in the general population in Japan, but they did not show that the precise classification of heart disease and other vascular diseases. Recently, our study has shown that psychiatric inpatients have increased silent brain infarction (SBI) and cerebral infarctions compared with Japanese healthy controls, accompanied with high prevalence of diabetes and low HDL-cholesterolemia [13]. These results suggest that cerebral incidents are also important in quality of life in psychiatric patients of Japan.

Psychiatric patients of our previous study were hospitalized patients who were diagnosed as schizophrenic group 69.1%, mood disorders group 18.4% and others 12.5% [13]. Schizophrenia and mood disorders are primary psychiatric diseases. Atypical antipsychotics are the major medication used to treat schizophrenia [9], and anti-depressants, mood-stabilizers and atypical antipsychotics are used to treat mood disorders. Therefore, it is possible that there are different metabolic changes in patients with schizophrenia and mood disorders. This is an essential point to plan the lowering the incidence of lifestyle-related diseases and cardiovascular diseases in each psychiatric patient with schizophrenia or mood disorders.

In this present study, we investigated the similarities and differences of lifestyle-related diseases between schizophrenia and mood disorders and also metabolic differences between patients with schizophrenia or mood disorders, and each standard in Japan. Decreased renal function is reported in hospitalized patients with female mood disorders.

## Methods

### Study design and study subjects

This cross-sectional observational study was performed from January 2015 to December 2017 at Kohnodai Hospital, National Center for Global Health and Medicine. Study participants were 189 Japanese hospitalized patients (82 males and 107 females) in the Psychiatry Department at Kohnodai Hospital. The diagnosis of psychiatric disorder was established as follows. Trained psychiatrists carried out neurological examinations and a diagnostic interview of the patients and reviewed information from the patients’ relatives. They excluded organic mental disorders and mental and behavioral disorders due to psychoactive substance use. A diagnosis was made using the ICD-10 classification of mental and behavioral disorders. Then, several psychiatrists discussed the assessment of the diagnosis and treatments in every patient at the conference opening every week. We then classified participants by schizophrenia group (F2 group, schizophrenia (F20), acute and transient psychotic disorders (F23) and schizoaffective disorders (F25)), mood disorders group (F3 group, bipolar affective disorder (F31), depressive episode (F32) and recurrent depressive disorder (F33)) and other mental disorders (Alzheimer’s disease, stimulant psychosis and somatoform disorders).

The study protocol was approved by the Ethics Committees of Chiba University (No. 182) and the National Center for Global Health and Medicine (No. 1837). All participants were provided with a written informed consent form, and explanation and participation agreement were performed in accordance with the Declaration of Helsinki principles.

### Diagnosis of somatic diseases in study participants

The definition of hypertension was above 140 mmHg of systolic blood pressure and/or above 90 mmHg of diastolic blood pressure [14]. Diabetes mellitus was defined as HbA1c over 6.5% and fasting plasma glucose (FPG) over 126 mg/dl [15]. High LDL-cholesterolemia (fasting serum LDL-cholesterol (LDL-C) ≥ 140 mg/dl) or low HDL-cholesterolemia (fasting serum HDL-cholesterol (HDL-C) < 40 mg/dl) or hypertriglyceridemia (fasting serum triglyceride (TG) ≥ 150 mg/dl) were described as dyslipidemia [16]. Patients were also counted as hypertension, diabetes or dyslipidemia if they used anti-hypertensive (Ca antagonists, angiotensin converting enzyme inhibitors, angiotensin receptor blockers, diuretics and β-blockers) or hypoglycemic (insulins, glucagon-like peptide-1 receptor agonists, biguanides, sulfonylureas, α-glucosidase inhibitors, thiazolidines, dipeptidyl peptidase-4 inhibitors and sodium glucose transporter-2 inhibitors) or anti-dyslipidemic drugs (statins, fibrates and ezetimibe), respectively. The diagnosis of metabolic syndrome (Met-S) was followed according to the definition of the Japan Society for the Study of Obesity. Met-S was diagnosed when waist circumference (male ≥ 85 cm, female ≥ 90 cm) plus two of the following criteria are met: high blood pressure (≥ 130/85), reduced HDL-C (< 40 mg/dl) and/or raised TG (≥ 150 mg/dl), and raised fasting hyperglycemia (≥ 110 mg/dl) [17]. Cerebral infarction was diagnosed by the presence of neurological symptoms and signs corresponding to brain imaging. The definition of smokers was patients who smoked until 1 month before admission.

### Data collections

Information on patients’ demographic data and medical history were obtained from their medical records. Body mass index (BMI) was calculated by their height and weight. Waist circumference was measured at a level midway between the lowest rib and the iliac crest. Hospital staff measured blood pressure on the right arm of a patient before breakfast. Blood samples were obtained from patients after 12 h starvation. Total cholesterol (TC) and TG were assayed by enzymatic method and HDL-C was by direct method. LDL-C was calculated by Friedewald formula from TC, TG and HDL-C (TC-TG/5-HDL-C) and non-HDL cholesterol (non-HDL-C) was TC minus HDL-C. HbA1c was measured by the high performance liquid chromatography (HPLC) method, and fasting plasma glucose and creatinine were by enzymatic method. Estimated glomerular filtration rate (eGFR) was calculated by serum creatinine level, age and gender.

### Brain imaging

The magnetic resonance imaging (MRI) examinations were performed in 5 mm thickness with 2 mm slice gap using a 1.5 Tesla MRI system (Siemens Magnetom Symphony). Patients underwent T1- and T2-weighted MRI and fluid-attenuated inversion recovery (FLAIR) of the brain as described by Yoshida et al. [18]. FLAIR images were used to distinguish infarcts from dilated perivascular spaces. We diagnosed silent brain infarction (SBI) as follows: (1) spotty area ≥ 3 mm in diameter showing high density in T2 and FLAIR images and low density in T1 image, (2) lack of neurological signs explained by MRI lesions, (3) no medical history of clinical symptoms of stroke [19]. 790 elderly volunteers (330 females and 460 males, mean age 61.0 years old, range 40–88) were used as control of SBI [20]. All of 790 volunteers were living independently at home without apparent history of stroke or dementia.

### Statistical analysis

When we compared physical and metabolic differences between F2 group and F3 group, first we used *t*-test in quantitative variables and Fisher’s exact test in qualitative variables without any adjustments. Next, we estimated age-adjusted mean differences and Odds ratios between F2 and F3 groups, using analysis of covariance (ANCOVA) and logistic regression analysis, respectively. Quantitative variables were BMI, waist circumstance, systolic blood pressure, diastolic blood pressure, LDL-C, HDL-C, triglyceride, non-HDL-C, fasting blood glucose, HbA1c, serum creatinine and eGFR. Qualitative variables were prevalence of hypertension, diabetes mellitus, high LDL-cholesterolemia, low HDL-cholesterolemia, hypertriglyceridemia, metabolic syndrome, smoking, and ratios of SBI and cerebral infarction, eGFR < 60 ml/min and albuminuria.

In comparisons between patients with schizophrenia or mood disorders and each standard, we quoted ‘The National Health and Nutrition Survey in Japan 2015’ produced by the Japanese Ministry of Health, Labor and Welfare [21] as the healthy Japanese standard. This survey was performed in 6655 persons (3064 were male, 3591 were female) chosen at random from all districts of Japan at November in 2015. Age of target persons was distributed from 1 to over 70 years old. For albuminuria, the data of the Takahata study was used [22].

Means or ratios in the standard group were calculated by adjusting sex and age configuration to patient group. Next, we estimated means or ratios with 95% of confidence intervals (CIs) of the patient group. Then we compared characteristics of F2 group or F3 group with those of each standard group. All analyses were conducted using a statistical software (SAS Analytics Pro Academic Suite; SAS Institute Inc.)

## Results

### Profile of study participants

Table 1 shows the profile of study patients. Schizophrenia group (F2 group) were 144 persons, and mood disorders group (F3 group) were 45. The ratio of male to female was 1:1.32 (62 persons: 82 persons) in F2 group, and was 1:1.25 (20 persons: 25 persons) in F3 group, indicating a similar ratio of male to female in both groups. The average age was approximately 13 years older in F3 group than in F2 group.

**Table 1:** Profile of study participants

	F2	F3
Cases (*n*)	144	45
Male	62	20
Female	82	25
Age, years (mean ± SD)	49.8 ± 12.3	62.6 ± 12.6
Male	46.0 ± 10.5	60.8 ± 11.3
Female	52.6 ± 12.8	64.1 ± 13.6
Diagnosis		
Schizophrenia group (F2)	144	
Schizophrenia (F20)	135	
Acute and transient psychotic disorders (F23)	7	
Schizoaffective disorders (F25)	2	
Mood disorders group (F3)		45
Bipolar affective disorder (F31)		30
Depressive episode (F32)		2
Recurrent depressive disorder (F33)		13

### Comparisons of physical characteristics and disorders between study participants of F2 group and F3 group

First, we compared physical characteristics and disorders between F2 and F3 study participants directly (Table 2). Significant difference between F2 and F3 groups was only observed in the prevalence of smoking by adjustment with age (*p* = 0.0148). Age-adjusted analysis showed no significant differences of BMI, blood pressure, the prevalence of hypertension and high LDL-cholesterolemia, and the ratio of SBI and cerebral infarction, in spite of their differences by simple analysis without age-adjustment.

**Table 2:** Comparisons of physical characteristics and disorders between F2 and F3 study participants

	F2			F3			F2 vs F3			
	*n*	Mean or ratio	SD	*n*	Mean or ratio	SD	T-test or Fisher’s exact test *p*	Age-adjusted		
								*p*	Mean differences (95% CI)	Odds ratios (95% CI)
Body mass index (BMI, kg/m^2^)	130	24.1	4.9	43	22.9	5	N.S.	N.S.	0.53 (− 1.3 to 2.4)	
Male	56	24.7	4.9	19	21.7	3.8	0.0018	N.S.	1.8 (− 1.0 to 4.7)	
Female	74	23.6	4.8	24	23.8	5.7	N.S.	N.A.		
Waist circumference (cm)	119	85.2	13.6	39	83.6	13.7	N.S.	N.A.		
Male	51	88.5	13.2	16	82.8	12.1	N.S.	N.A.		
Female	68	82.6	13.5	23	84.2	14.9	N.S.	N.A.		
Systolic blood pressure (mmHg)	144	125.3	16	45	131.6	18.2	0.026	N.S.	− 3.3 (− 9.4 to 2.7)	
Male	62	127.5	16.2	20	131.5	18.6	N.S.	N.A.		
Female	82	123.5	15.8	25	131.7	18.3	0.031	N.S.	− 3.2 (− 10.8 to 4.3)	
Diastolic blood pressure (mmHg)	144	77.2	11.4	45	77.0	11.1	N.S.	N.A.		
Male	62	80.4	10.9	20	77.1	9.7	N.S.	N.S.	− 0.3 (− 6.4 to 5.9)	
Female	82	74.8	11.3	25	77.0	12.4	N.S.	N.A.		
Hypertension	144	0.271		45	0.467		0.017	N.S.		0.6 (0.28 to 1.28)
Male	62	0.355		20	0.450		N.S.	N.A.		
Female	82	0.207		25	0.480		0.011	N.S.		0.47 (0.17 to 1.32)
Diabetes mellitus	144	0.160		45	0.156		N.S.	N.S.		2.00 (0.77 to 5.65)
Male	62	0.190		20	0.100		N.S.	N.A.		
Female	82	0.130		25	0.200		N.S.	N.S.		1.20 (0.33 to 4.44)
High LDL-cholesterolemia	140	0.264		44	0.364		N.S.	N.S.		0.85 (0.39 to 1.89)
Male	60	0.283		19	0.211		N.S.	N.A.		
Female	80	0.250		25	0.480		0.045	N.S.		0.46 (0.17 to 1.27)
Low HDL-cholesterolemia	139	0.209		44	0.205		N.S.	N.A		
Male	59	0.322		19	0.316		N.S.	N.A		
Female	80	0.125		25	0.12		N.S.	N.A		
Hypertriglyceridemia	143	0.210		44	0.205		N.S.	N.A		
Male	62	0.258		19	0.158		N.S.	N.A		
Female	81	0.173		25	0.240		N.S.	N.A		
Metabolic syndrome	115	0.209		38	0.263		N.S.	N.A.		
Male	49	0.306		15	0.200		N.S.	N.A.		
Female	66	0.136		23	0.304		N.S.	N.S.		0.61 (0.17 to 2.13)
Prevalence of smoking	139	0.396		40	0.125		0.0011	0.0148*		3.62 (1.29 to 10.4)
Male	61	0.541		16	0.188		0.013	N.A.		
Female	78	0.282		24	0.083		N.S.	N.A.		
Ratio of SBI and cerebral infarction	141	0.234		44	0.477		0.0039	N.S.		1.06 (0.426 to 2.64)

First, we used t-test in quantitative variables and Fisher’s exact test in qualitative variables between F2 and F3 groups without any adjustments. Next, Age-adjusted mean differences were estimated using ANCOVA and Odds ratios were calculated using logistic regression analysis. CI is an abbreviation of confidence interval. N.A. means not applicable. N.S. means not significant (*p* ≥ 0.05). * is significantly different (*p* < 0.05) between F2 group and F3 group by age-adjusted analysis

Table 3 shows that female BMI was significantly higher in the F2 group compared with the age-adjusted Japanese standard population (the standard) but was lower in the male F3 group. Waist circumference and blood pressure were not significantly different in either group except lower diastolic blood pressure of male F3 group. We calculated the prevalence of hypertension, diabetes mellitus, dyslipidemia, metabolic syndrome and smoking in both study groups (Table 3). The ratio of low HDL-cholesterolemia was significantly higher in both groups than the standard. The ratio of diabetes was significantly high in the F2 group but was no significant difference in the F3 group compared with the standard. These results are in accordance with the increased ratio of metabolic syndrome in the F2 group. However, the ratios of high LDL-cholesterolemia and hypertension showed no significant differences in both groups compared with the standard except lower ratio of hypertension in female F2 group, and the ratio of hypertriglyceridemia was significantly lower in both male groups. The prevalence of smoking was significantly higher in the F2 group than the standard (Table 3).

**Table 3:** Differences of physical characteristics and disorders between F2 and F3 study participants and standard

	F2				F3			
	F2 patient group			Age-adjusted mean or ratio of standard group	F3 patient group			Age-adjusted mean or ratio of standard group
	*n*	Mean or ratio	95% CI		*n*	mean or ratio	95% CI	
Body mass index (BMI, kg/m^2^)	130	24.1*	23.2–24.9	23.1	43	22.9	21.3–24.4	23.2
Male	56	24.7	23.4–26.1	23.9	19	21.7*	19.9–23.5	23.8
Female	74	23.6*	22.5–24.7	22.5	24	23.8	21.3–26.2	22.8
Waist circumference (cm)	119	85.2	82.7–87.6	82.9	39	83.6	79.2–88.1	84.3
Male	51	88.5	84.8–92.3	86.0	16	82.8	76.4–89.3	86.7
Female	68	82.6	79.4–85.9	80.5	23	84.2	77.7–90.6	82.6
Systolic blood pressure (mmHg)	144	125.3	122.6–127.9	126	45	131.6	126.1–137.1	132.8
Male	62	127.5	123.4–131.6	128.4	20	131.5	122.8–140.2	134.9
Female	82	123.5	120.1–127.0	124.2	25	131.7	124.1–139.3	131.2
Diastolic blood pressure (mmHg)	144	77.2	75.3–79.1	79.0	45	77.0	73.7–80.4	80.1
Male	62	80.4	77.6–83.1	82.0	20	77.1*	72.5–81.6	82.7
Female	82	74.8	72.3–77.2	76.7	25	77.0	71.9–82.1	78.1
Hypertension	144	0.271	0.200–0.351	0.349	45	0.467	0.317–0.621	0.555
Male	62	0.355	0.237–0.487	0.367	20	0.450	0.231–0.685	0.590
Female	82	0.207*	0.126–0.311	0.336	25	0.480	0.278–0.687	0.527
Diabetes mellitus	144	0.160*	0.104–0.230	0.089	45	0.156	0.065––0.295	0.145
Male	62	0.194	0.104–0.314	0.105	20	0.100	0.012–0.317	0.193
Female	82	0.134	0.069–0.227	0.077	25	0.200	0.068–0.407	0.106
High LDL-cholesterolemia	140	0.264	0.193–0.345	0.241	44	0.364	0.224–0.522	0.252
Male	60	0.283	0.175–0.414	0.237	19	0.211	0.061–0.456	0.215
Female	80	0.250	0.160–0.359	0.244	25	0.480	0.278–0.687	0.280
Low HDL-cholesterolemia	139	0.209*	0.144–0.286	0.073	44	0.205*	0.098–0.353	0.092
Male	59	0.322*	0.206–0.456	0.134	19	0.316	0.126–0.566	0.155
Female	80	0.125*	0.062–0.218	0.028	25	0.120	0.026–0.312	0.044
Hypertriglyceridemia	143	0.210*	0.146–0.286	0.331	44	0.205*	0.098–0.353	0.354
Male	62	0.258*	0.155–0.385	0.446	19	0.158*	0.034–0.396	0.436
Female	81	0.173	0.098–0.273	0.243	25	0.240	0.094–0.451	0.292
Metabolic syndrome	115	0.209*	0.141–0.294	0.123	38	0.263	0.130–0.421	0.190
Male	49	0.306*	0.191–0.459	0.180	15	0.200	0.041–0.457	0.283
Female	66	0.136	0.062–0.236	0.068	23	0.304*	0.132–0.529	0.125
Prevalence of smoking	139	0.396*	0.314–0.482	0.237	40	0.125	0.042–0.268	0.176
Male	61	0.541*	0.409–0.669	0.395	16	0.188	0.041–0.457	0.326
Female	78	0.282*	0.186–0.395	0.113	24	0.083	0.010–0.270	0.076
Ratio of SBI and cerebral infarction	141	0.234*	0.155–0.297	0.141	44	0.477*	0.325–0.633	0.304

Means or ratios in the standard group were calculated by adjusting sex and age configuration to patient group. Next, we estimated means or ratios with 95% of confidence intervals (CIs) of the patient group. *means significantly different (*p* < 0.05) between patient group and standard group

As far as brain MRI, 185 patients agreed to undergo brain imaging. 2 and 3 patients were diagnosed as cerebral infarction in F2 and F3 groups, respectively, by the presence of neurological symptoms and signs inferred from brain imaging. The ratios of SBI plus cerebral infarction were 0.234 and 0.477 in the F2 and F3 groups, respectively, and significantly higher than the age-adjusted ratio of each control (Table 3).

### Comparisons of metabolic characteristics between study participants of F2 group and F3 group

Tables 4 and 5 show serum blood levels of lipid and glucose, and renal function. First, we compared metabolic characteristics between F2 and F3 study participants directly (Table 4). Female eGFR in F3 group was significantly lower than female F2 group by age-adjustment (*p* = 0.0225). Age-adjusted analysis showed no significant differences of HbA1c, serum creatinine and the ratio of eGFR < 60 ml/min, in spite of their differences by simple analysis without age-adjustment.

**Table 4:** Comparisons of metabolic characteristics between F2 and F3 study participants

	F2			F3			F2 vs F3			
	*n*	Mean or ratio	SD	*n*	Mean or ratio	SD	T-test or Fisher’s exact test p=	Age-adjusted		
								*p*	Mean differences (95% CI)	Odds ratios (95% CI)
*Lipid metabolism*										
LDL-C (mg/dl)	139	113.6	38.2	44	113.5	33.9	N.S.	N.A.		
Male	59	107.3	96.4	19	106.9	95.7	N.S.	N.A.		
Female	80	118.3	34.7	25	118.6	39.9	N.S.	N.A.		
HDL-C (mg/dl)	139	53.9	17.8	44	55.6	17.4	N.S.	N.A.		
Male	59	48.7	13.7	19	49	15.4	N.S.	N.A.		
Female	80	57.7	19.6	25	60.5	17.5	N.S.	N.A.		
Triglyceride (mg/dl)	143	115.5	83.0	44	112	63.6	N.S.	N.A.		
Male	62	126.2	94.3	19	111	76.9	N.S.	N.A.		
Female	81	107.4	72.8	25	113.6	52.9	N.S.	N.A.		
Non-HDL-C (mg/dl)	139	137	43.1	44	135.9	38.0	N.S.	N.A.		
Male	59	133.7	48.3	19	128.9	28.2	N.S.	N.A.		
Female	80	139.5	39.0	25	141.3	43.8	N.S.	N.A.		
*Glucose metabolism*										
Fasting blood glucose (mg/dl)	144	117.7	43.4	45	131.5	76.2	N.S.	N.S.	− 7.2 (− 26.6 to 12.3)	
Male	62	117.7	47.7	20	126.8	91.0	N.S.	N.A.		
Female	82	117.7	40.2	25	135.2	63.7	N.S.	N.S.	− 11.1 (− 33.5 to 11.4)	
HbA1c (%)	144	5.82	0.81	45	6.02	1.18	N.S.	N.S.	− 0.01 (− 0.34 to 0.32)	
Male	62	5.91	1.01	20	5.97	1.62	N.S.	N.A.		
Female	82	5.75	0.60	25	6.06	0.69	0.029	N.S.	− 0.12 (− 0.40 to 0.16)	
*Renal function*										
Serum creatinine (mg/dl)	144	0.696	0.234	45	0.778	0.250	0.045	N.A.		
Male	62	0.788	0.218	20	0.877	0.307	N.S.	N.A.		
Female	82	0.626	0.223	25	0.698	0.160	N.S.	N.A.		
eGFR (ml/min)	144	87.1	22.8	45	72.4	21.4	0.0002	0.0139*	10.3 (2.1 to 18.5)	
Male	62	90.6	22.9	20	77.1	24.9	0.027	N.S.	9.9 (− 4.0 to 23.8)	
Female	82	84.4	22.5	25	68.7	17.8	0.002	0.0225*	12 (1.7 to 22.3)	
Ratio of eGFR < 60 ml/min	144	0.076		45	0.244		0.006	N.S.		0.45 (0.16–1.2)
Male	62	0.048		20	0.15		N.S.	N.S.		0.48 (0.07–3.7)
Female	82	0.098		25	0.32		0.011	N.S.		0.36 (0.11–1.2)
Ratio of albuminuria (30 mg/g creatinine <) (40 years <)	93	0.043		33	0.152		N.S.	N.S.		0.34 (0.08–1.41)
Male	37	0.027		14	0.071		N.S.	N.S.		0.53 (0.03–10.1)
Female	56	0.054		19	0.211		N.S.	N.S.		0.27 (0.05–1.44)

First, we used t-test in quantitative variables and Fisher’s exact test in qualitative variables between F2 and F3 groups without any adjustments. Next, Age-adjusted mean differences were estimated using ANCOVA and Odds ratios were calculated using logistic regression analysis. CI is an abbreviation of confidence interval. N.A. means not applicable. N.S. means not significant (*p* ≥ 0.05). * is significantly different (*p* < 0.05) between F2 group and F3 group by age-adjusted analysis

**Table 5:** Differences of metabolic characteristics between F2 or F3 study participants and standard

	F2				F3			
	F2 patient group			Age-adjusted mean or ratio of standard group	F3 patient group			Age-adjusted mean or ratio of standard group
	*n*	Mean or ratio	95% CI		*n*	mean or ratio	95% CI	
*Lipid metabolism*								
LDL-C (mg/dl)	139	113.6	107.2–120.0	118.7	44	113.5	103.2–123.8	121.3
Male	59	107.3	99.4–118.3	118	19	106.9	95.7–118.0	116.4
Female	80	118.3	110.6–126.0	119.2	25	118.6	102.2–135.1	125
HDL-C (mg/dl)	139	53.9*	50.9–56.9	61.7	44	55.6	50.3–60.9	59.6
Male	59	48.7*	45.1–52.3	55.9	19	49	41.6–56.4	54.9
Female	80	57.7*	53.3–62.0	66	25	60.5	53.3–67.8	63
Triglyceride (mg/dl)	143	115.5*	101.8–129.3	138.2	44	112*	92.7–131.3	141.9
Male	62	126.2*	102.2-150.1	160.2	19	111*	73.0–147.0	165
Female	81	107.4	91.3–123.5	121.1	25	113.6	91.7–135.4	124.3
Non-HDL-C (mg/dl)	139	137.0*	129.8–144.3	145.2	44	135.9*	124.4–147.5	148.7
Male	59	133.7*	121.1–146.3	148.1	19	128.9*	115.3–142.5	147.4
Female	80	139.5	130.8–148.2	143.1	25	141.3	123.2–159.4	149.6
*Glucose metabolism*								
Fasting blood glucose (mg/dl)	144	117.7*	110.6–124.9	98.1	45	131.5*	108.6–154.4	101.6
Male	62	117.7*	105.6–129.8	95.5	20	126.8	84.2–169.4	101
Female	82	117.7*	108.9–126.5	98	25	135.2*	108.9–161.5	102.1
HbA1c (%)	144	5.82*	5.69–5.95	5.57	45	6.02*	5.67–6.38	5.66
Male	62	5.91*	5.65–6.17	5.53	20	5.97	5.21–6.73	5.71
Female	82	5.75*	5.62–5.88	5.6	25	6.06*	5.78–6.35	5.63
Renal function								
Serum creatinine (mg/dl)	144	0.696	0.657–0.734	0.733	45	0.778	0.703–0.853	0.736
Male	62	0.788*	0.733–0.843	0.865	20	0.877	0.733–1.020	0.85
Female	82	0.626	0.578–0.675	0.633	25	0.698	0.633–0.764	0.644
eGFR (ml/min)	144	87.1	83.3-90.8	85.2	45	72.4	66.0–78.8	77.3
Male	62	90.6	84.8–96.4	85.6	20	77.1	65.4–88.7	77
Female	82	84.4	79.5–89.4	84.8	25	68.7*	61.4–76.1	77.5
Ratio of eGFR < 60 ml/min	144	0.076	0.039–0.133	0.048	45	0.244	0.129–0.395	0.136
Male	62	0.048	0.010–0.135	0.035	20	0.150	0.032–0.379	0.135
Female	82	0.098	0.043–0.183	0.058	25	0.320*	0.150–0.535	0.137
Ratio of albuminuria (30 mg/g creatinine <) (40 years <)	93	0.043	0.012–0.107	0.100	33	0.152	0.051–0.319	0.122
Male	37	0.027	0.001–0.142	0.101	14	0.071	0.002–0.339	0.115
Female	56	0.054	0.011–0.149	0.099	19	0.211	0.061–0.456	0.127

Means or ratios in the standard group were calculated by adjusting sex and age configuration to patient group. Next, we estimated means or ratios with 95% of confidence intervals (CIs) of the patient group. *means significantly different (*p* < 0.05) between patient group and standard group

Patients in the F2 group had significantly lower levels of HDL-C and TG than the standard except TG in the females. In F3 group, TG in males was significantly low but TG in females and HDL-C were not significantly different from the standard. LDL-C levels were not significantly different in either group compared with the standard. Non-HDL-C level was significantly low in male F2 and F3 groups. FPG and HbA1c were significantly higher in the F2 group and female F3 group than the standard.

Chronic kidney disease (CKD) is recognized as one of the important risk factors of cardiovascular diseases. The definition of CKD is continuous decreased GFR (GFR < 60 ml/min) and/or albuminuria [23]. The mean level of eGFR was 68.7 ml/min (95% CI was 61.4–76.1) in female F3 group and significantly lower than the age-adjusted mean of the standard (77.5 ml/min) (Table 5). We also calculated the ratio of eGFR lower than 60 ml/min. The ratio of eGFR lower than 60 ml/min was significantly higher in the female F3 group than the female standard, suggesting increased CKD. We also checked the urine albumin in subjects over 40 years old, another criterion of CKD definition [23]. The ratio of albuminuria tended to higher in the female F3 group than the female standard.

eGFR levels were not significantly different in the F2 group and male F3 group compared with each standard group (Table 5).

### Psychiatric medications administered to study participants

In the F2 group, 76.6% of the psychiatric medications used is atypical antipsychotics, 21.4% is typical and 1.9% is other psychotropic medications. Among administered medicines in the F3 group, atypical antipsychotics accounted for 54.7%, antidepressants and mood stabilizers 34.0% and typical antipsychotics 9.4% (Additional file 1). Therefore, most of the schizophrenic patients were treated with antipsychotics, but mood disorders patients were treated with various psychotropic medications containing atypical antipsychotics, antidepressants and mood stabilizers.

## Discussion

The present study shows that silent brain infarction and cerebral infarction were increased in patients with both schizophrenia and mood disorders, accompanied by glucose and lipid disorders. Differences between inpatients with schizophrenia and those with mood disorders were observed in renal function and prevalence of smoking.

Low HDL-cholesterolemia is defined by a serum level below 40 mg/dl [16]. We observed low HDL-cholesterolemia in this study. There are many reports that HDL-C level is low in medicated schizophrenia [24, 25]. Sagud et al. [26] reported that HDL-C was decreased in patients with bipolar affective disorder and major depressive disorder, but Peng et al. [27] showed that serum HDL-C level was elevated in those with major depressive disorder excluding psychiatric medications. HDL-C levels are decided by not only lifestyle-related diseases, but using psychotropic medications. Antipsychotics are known to lower serum HDL-C levels [24, 25]. It is reported that antidepressants are associated with weight gain but have fewer effects on lipid and glucose metabolism [28, 29]. Usually, patients with schizophrenia use atypical or typical antipsychotics, but those with mood disorders are administered by not only antidepressants or mood stabilizers but atypical antipsychotics. Therefore, it is possible to think that drug variation is one of the reasons why HDL-C in mood disorders is different in these reports.

Levels of serum TG and non-HDL-C were significantly low compared with the standard in both male F2 and F3 groups, and LDL-C was almost the same as the standard in both groups. Kingsbury et al. [30] described lower levels of serum TG in ziprasidone-treated patients with schizophrenia. However, there were reports of high serum TG or LDL-C levels in schizophrenic patients [24, 31]. Furthermore, Sugai et al. [32] showed that the levels of TG and LDL-C were higher in outpatients with schizophrenia than in inpatients. In patients with mood disorders, serum lipid levels are also variable. Hummel et al. [33] reported that serum TG level was higher and LDL-C was lower than controls. Lehto et al. [34] showed that serum TG and LDL-C were increased compared with controls. There was also a report that serum LDL-C was not changed in mood disorders patients [35]. These differences of TG and LDL-C levels might be dependent on the situation of the patients such as outpatients or inpatients, and drugs administered.

It is reported that the increased ratio of diabetes and high FPG are observed in patients with both schizophrenia and mood disorders, in accordance with high FPG and HbA1c in patients of the F2 group and female F3 group in our study. Stubbs et al. [36] described that schizophrenic patients had at least double the risk of diabetes by meta-analysis. Newcomer [37], Wysokinski et al. [38] and Vancampfort et al. [39] showed that the prevalence of diabetes or the level of FPG was high in patients with schizophrenia and those with mood disorders. It is well known that poor lifestyle is one of the major causes of increased diabetes. There are also many reports that antipsychotics, especially atypical ones have side effects on glucose metabolism [10, 40]. Furthermore, Ji et al. [41] also reported the genetic overlap between type 2 diabetes and major depressive disorders. Causes of increased diabetes or FPG in patients with schizophrenia and mood disorders remain to be elucidated.

There are several reports concerning psychiatric disorders and renal function. Tzeng et al. [42] reported that schizophrenia is associated with a 25% increase in the risk of developing CKD for 3 years follow up period. There are other reports that the prevalence of CKD is not different between schizophrenic patients and control [4], and the incidence of end-stage renal disease is low in schizophrenic patients [43]. Rej [44] and Kessing [45] showed that Lithium, mood stabilizing treatment for bipolar disorder, had an effect on renal function and induced CKD. This F3 group contained 8% Lithium users. eGFR in the female F3 group excluding Lithium users was 67.2 ml/min and the standard was 77.2 ml/min, which was significantly different, indicating that Lithium is not correlated with CKD in the F3 group in our study. We need to have an extended follow up renal function in these F2 and F3 patients.

As risk factors of developing CKD, there are lifestyle-related diseases, e.g., hypertension, diabetes, dyslipidemia, obesity and smoking [46]. Table 4 shows that eGFR was significantly lower in F3 group than F2 group by adjustment for age (*p* = 0.0139). By adjustments for age and prevalence of hypertension, there was significant difference between F2 group and F3 group (*p* = 0.0232). However, we have no significant differences by adjustment for smoking and diabetes (*p* = 0.2093 and *p* = 0.6309, respectively). These results mean that there is significant difference of eGFR between F2 and F3 groups which cannot be explained by age and hypertension. The causes of lowering eGFR in mood disorders remain to be clarified.

The ratios of SBI and cerebral infarction were higher in both F2 and F3 groups, compared with each control [20], indicating the increasing tendency to cerebrovascular changes. We did not detect significant differences between F2 and F3 groups by age-adjustment. There are reports about the relation of cerebrovascular changes to major depression. Yanai et al. [47] reported that patients with depression and SBI were more likely to develop psychiatric and neurological disorders than those with depression without SBI. A 10 years follow-up study showed that the presence of SBI is associated with a relatively poor prognosis in patients with depression [48]. The term ‘vascular depression’ has been used to describe depression occurring later in life and characterized by cerebral changes related to depression onset. The mean age of the F3 group is 62.6 years old. Therefore, a part of SBI positive depressive patients might be vascular depression.

This study also has some limitations. First, it was a cross-sectional study. It is impossible to clarify the cause-effect relationship between diabetes, hypertension, lipid, eGFR and SBI. Second, patients in our study were inpatients. It is probable that lifestyle-related diseases and metabolic profiles are not same in inpatients and outpatients [49]. Third, the number of mood disorders group is 45, about one-third of schizophrenia group. It may cause to miss the significant difference which can be detected if patient numbers are same in both groups. Fourth, the number of mood disorders group is small and much different from schizophrenic group to analyze the characteristics of bipolar affective disorder and major depressive disorder, separately.

## Conclusions

In conclusion, we found that patients with schizophrenia or mood disorders had increased ratio of SBI and cerebral infarction accompanied with glucose and lipid disorders in this study. Patients with mood disorders had decreased eGFR and prevalence of smoking compared with those with schizophrenia.

## Supplementary information

**Additional file 1**

## Data Availability

Not applicable.

## Abbreviations

ANCOVA: Analysis of covariance
BMI: Body mass index
CI: Confidence interval
CKD: Chronic kidney disease
eGFR: Estimated glomerular filtration rate
FLAIR: Fluid-attenuated inversion recovery
FPG: Fasting plasma glucose
HDL: High-density lipoprotein
HPLC: High performance liquid chromatography
LDL: Low-density lipoprotein
MRI: The magnetic resonance imaging
N.A.: Not applicable
N.S.: Not significant
SBI: Silent brain infarction
TC: Total cholesterol
TG: Triglyceride

## References

[1] Hennekens CH, Hennekens AR, Hollar D (2005). Schizophrenia and increased risks of cardiovascular disease. Am Heart J.

[2] Hjorthoj C, Sturup AE, McGrath JJ (2017). Years of potential life lost and life expectancy in schizophrenia: a systematic review and meta-analysis. Lancet Psychiatry..

[3] Kondo S, Kumakura Y, Kanehata A (2017). Premature deaths among individuals with severe mental illness after discharge form long-term hospitalisation in Japan: a naturalistic observation during a 24-year period. BJPsych open..

[4] Crump C, Winkleby MA, Sundquist K, Sundquist J (2013). Comorbidities and mortality in persons with schizophrenia: a Swedish National Cohort Study. Am J Psychiatry.

[5] Smith DJ, Langan J, McLean G, Guthrie B, Mercer SW (2013). Schizophrenia is associated with excess multiple physical-health comorbidities but low levels of recorded cardiovascular disease in primary care: cross-sectional study. BMJ Open.

[6] Okumura Y, Ito H, Kobayashi M (2010). Prevalence of diabetes and antipsychotic prescription patterns in patients with schizophrenia: a nationwide retrospective cohort study. Schizophrenia Res..

[7] Sugawara N, Yasui-Furukori N, Sato Y (2010). Prevalence of metabolic syndrome among patients with schizophrenia in Japan. Schizophrenia Res..

[8] Davidson S, Judd F, Jolley D (2001). Cardiovascular risk factors for people with mental illness. Aust NZJ Psychiatry..

[9] Halfdanarson O, Zoega H, Aagaard L (2017). International trends in anti-psychotic use: a study in 16 countries, 2005-2014. Eur Neuropsychopharmacol.

[10] Newcomer JW, Rantner RE, Eriksson JW (2009). A 24-week, multicenter, open-label, randomized study to compare changes in glucose metabolism in patients with schizophrenia receiving treatment with olanzapine, quetiapine, or risperidone. J Clin Psychitry..

[11] Meyer JM, Koro CE (2004). The effects of antipsychotic therapy on serum lipids: a comprehensive review. Schizophrenia Res..

[12] Saku M, Tokudome S, Ikeda M (1995). Mortality in psychiatric patients, with a specific focus on cancer mortality associated with schizophrenia. Int J Epidemiol.

[13] Kanzaki T, Uju Y, Sekine K (2015). Increased silent brain infarction accompanied with high prevalence of diabetes and dyslipidemia in psychiatric inpatients: a cross-sectional study. Prim Care Companion CNS Disord..

[14] The Japanese Society of Hypertension Committee for Guidelines for the Management of Hypertension (2014). The Japanese Society of Hypertension Guidelines for the Management of Hypertension (JSH2014). Hypertens Res.

[15] Seino Y, Nanjo K, Tajima N, Kadowaki T, Kashiwagi A, Araki E, Ito C, Inagaki N, Iwamoto Y, Kasuga M, Hanafusa T (2010). The committee of the Japan Diabetic Society on the diagnostic criteria of diabetes mellitus. Report of the committee on the classification and diagnostic criteria of diabetes mellitus. Diabetol Int..

[16] Committee for Epidemiology and Clinical Management of Atherosclerosis (2013). Diagnostic criteria for dyslipidemia. J Atheroscler Thromb..

[17] Matuzawa Y (2005). Metabolic syndrome-definition and diagnostic criteria in Japan. J Atheroscler Thromb..

[18] Yoshida M, Tomitori H, Machi Y (2009). Acrolein, IL-6 and CRP as markers of silent brain infarction. Atherosclerosis..

[19] Vermeer SE, Hollander M, van Dijk EJ (2003). Silent brain infarcts and white matter lesions increase stroke risk in the general population: the Rotterdam Scan Study. Stroke.

[20] Yoshida M, Higashi K, Kobayashi E (2010). Correlation between images of silent brain infarction, carotid atherosclerosis and white matter hyperintensity, and plasma levels of acrolein, IL-6 and CRP. Atherosclerosis..

[21] Ministry of Health, Labor and Welfare, Japan. The National Health and Nutrition Survey in Japan. 2015.

[22] Konta T, Hao Z, Abiko H (2006). Prevalence and risk factor analysis of microalbuinuria in Japanese general population: the Takahata study. Kidney Int.

[23] Levey AS, Eckardt KU, Tsukamoto Y (2005). Definition and classification of chronic kidney disease: a position statement from kidney disease: improving global outcomes (KDIGO). Kidney Int.

[24] Murashita M, Inoue T, Kusumi I (2007). Glucose and lipid metabolism of long-term risperidone monotherapy in patients with schizophrenia. Psychiatry Clin Neurosci.

[25] Sasaki J, Kumagae G, Sata T, Kuramitsu M, Arakawa K (1984). Decreased concentration of high density lipoprotein cholesterol in schizophrenic patients treated with phenothiazines. Atherosclerosis..

[26] Sagud M, Mihaljevic-Peles A, Pivac N (2009). Lipid levels in female patients with affective disorders. Psychiatry Res.

[27] Peng Y-F, Xiang Y, Wei Y-S (2016). The significance of routine biochemical markers in patients with major depressive disorder. Sci Rep..

[28] Himmerich H, Minkwitz J, Kirkby KC (2015). Weight gain and metabolic changes during treatment with antipsychotics and antidepressants. Endocr Metab Immune Disord Drug Targets.

[29] Hennings JM, Schaaf L, Fulda S (2012). Glucose metabolism and antidepressant medication. Curr Pharm Des.

[30] Kingsbury SJ, Fayek M, Trufasiu D (2001). The apparent effects of ziprasidone on plasma lipids and glucose. J Clin Psychiatry.

[31] Tschoner A, Engl J, Rettenbacher M (2009). Effects of six second generation antipsychotics of body weight and metabolism- risk assessment and results from a prospective study. Pharmacopsychiatry..

[32] Sugai T, Suzuki Y, Yamazaki M (2016). High prevalence of obesity, hypertension, hyperlipidemia, and diabetes mellitus in Japanese outpatients with schizophrenia: a nationwide survey. PLoS ONE.

[33] Hummel J, Westphal S, Weber-Hamann B (2011). Serum lipoproteins improve after successful pharmacologic antidepressant treatment: a randomized open-label prospective trial. J Clin Psychiatry.

[34] Lehto SM, Niskanen L, Tolmunen T (2010). Low serum HDL-cholesterol levels are associated with long symptom duration in patients with major depressive disorder. Psychiatry Clin Neurosci.

[35] Nunes SOV, de Melo LGP, de Castro MRP (2015). Atherogenic index of plasma and atherogenic coefficient are increased in major depression and bipolar disorder, especially when comorbid with tobacco use disorder. J Affect Disord.

[36] Stubbs B, Vancampfort D, De Hert M, Mitchell AJ (2015). The prevalence and predictors of type two diabetes mellitus in people with schizophrenia: a systematic review and comparative meta-analysis. Acta Psychiatr Scand.

[37] Newcomer JW (2006). Medical risk in patients with bipolar disorder and schizophrenia. J Clin Psychiatry.

[38] Wysokinski A, Strzelecki D, Kloszewska I (2015). Levels of triglycerides, cholesterol, LDL, HDL and glucose in patients with schizophrenia, unipolar depression and bipolar disorder. Diabetes Metab Syndr..

[39] Vancampfort D, Mitchell AJ, De Hert M (2015). Type 2 diabetes in patients with major depressive disorder: a meta-analysis of prevalence estimates and predictors. Depress Anxiety..

[40] Akhtar S, Kelly C, Gallagher A, Petrie JR (2004). Newer antipsychotic agents, carbohydrate metabolism and cardiovascular risk. Br J Diabetes Vasc Dis..

[41] Ji H-F, Zhuang Q-S, Shen L (2016). Genetic overlap between type 2 diabetes and major depressive disorder identified by bioinformatics analysis. Oncotarget..

[42] Tzeng N-S, Hsu Y-H, Ho S-Y (2015). Is schizophrenia associated with an increased risk of chronic kidney disease? A nationwide matched-cohort study. BMJ Open..

[43] Hsu Y-H, Cheng J-S, Ouyang W-C (2015). Lower incidence of end-stage renal disease but suboptimal pre-dialysis renal care in schizophrenia: a 14-year nationwide cohort study. PLoS ONE.

[44] Rej S, Elie D, Mucsi I, Looper KJ, Segal M (2015). Chronic kidney disease in Lithium-treated older adults: a review of epidemiology, mechanisms, and implications for the treatment of late-life mood disorders. Drugs Aging.

[45] Kessing LV, Gerds TA, Feldt-Rasmussen B, Andersen PK, Licht RW (2015). Use of lithium and anticonvulsants and the rate of chronic kidney disease. A nationwide population-based study. JAMA Psychiatry..

[46] Yamagata K, Ishida K, Sairenchi T (2007). Risk factor for chronic kidney diseases in a community-based population: 10-year follow-up study. Kidney Int.

[47] Yanai I, Fujikawa T, Horiguchi J (1998). The 3-year course and outcome of patients with major depression and silent cerebral infarction. J Affect Disord.

[48] Yamashita H, Fujikawa T, Takami H (2010). Long-term prognosis of patients with major depression and silent cerebral infarction. Neuropsychobiology..

[49] Uju Y, Kanzaki T, Yamasaki Y (2020). Metabolic changes of Japanese schizophrenic patients transferred from hospitalization to outpatients. Global Health Med..

