# Supplementary material for: A cross-sectional study on metabolic similarities and differences between inpatients with schizophrenia and those with mood disorders
Source: Ann Gen Psychiatry. 2020 Sep 22;19:53. doi: 10.1186/s12991-020-00303-5 (PMC7510094; doi:10.1186/s12991-020-00303-5)
Supplement: Supplementary file 1 — Additional file 1 [file 12991_2020_303_MOESM1_ESM.docx]

| Additional file 1 Psychiatric medications mainly administered to study participants | | |
| --- | --- | --- |
|  | F2 | F3 |
| no. of patients | 144 | 45 |
| Atypical antipsychotic | 118 (76.6%) | 29 (54.7%) |
| risperidone | 52 |  |
| olanzapine | 36 | 13 |
| quetiapine | 4 | 12 |
| clozapine | 8 |  |
| aripirazol | 6 | 4 |
| paliperidone | 6 |  |
| blonanserine | 4 |  |
| perospirone | 2 |  |
| Typical antipsycotic | 33 (21.4%) | 5 (9.4%) |
| haloperidol | 16 | 3 |
| zotepine | 6 | 1 |
| pipamperone | 5 |  |
| perphenazine (PZC) | 1 | 1 |
| timiperone | 3 |  |
| bromperidol | 2 |  |
| Others | 3 (1.94%) | 18 (34.0%) |
| antidepressant |  |  |
| mirtazapine |  | 7 |
| sulpiride |  | 1 |
| mood stabilizer |  |  |
| lithium carbonate | 1 | 4 |
| lamotrigine |  | 2 |
| valproate | 2 | 2 |
| carbamazepine |  | 2 |
| None | 0 | 1 (1.9%) |
